# Automated Tumor and Node Staging from Esophageal Cancer Endoscopic Ultrasound Reports: A Benchmark of Advanced Reasoning Models with Prompt Engineering and Cross-Lingual Evaluation

**DOI:** 10.3390/diagnostics16020215

**Published:** 2026-01-09

**Authors:** Xudong Hu, Lingde Feng, Bingzhong Jing, Linna Luo, Wencheng Tan, Yin Li, Xinyi Zheng, Xinxin Huang, Shiyong Lin, Huiling Wu, Longjun He

**Affiliations:** 1 Department of Endoscopy, Sun Yat-Sen University Cancer Center, State Key Laboratory of Oncology in South China, Collaborative Innovation Center for Cancer Medicine, Guangzhou 510060, China; 2 Department of Information Center, Sun Yat-Sen University Cancer Center, State Key Laboratory of Oncology in South China, Collaborative Innovation Center for Cancer Medicine, Guangzhou 510060, China

**Keywords:** Large Language Models, DeepSeek-R1, Esophageal cancer, TNM staging, endoscopic ultrasound, prompt engineering, cross-lingual evaluation, robustness

## Abstract

**Objectives:** To benchmark the performance of DeepSeek-R1 against three other advanced AI reasoning models (GPT-4o, Qwen3, Grok-3) in automatically extracting T/N staging from esophageal cancer endoscopic ultrasound (EUS) complex medical reports, and to evaluate the impact of language (Chinese/English) and prompting strategy (with/without designed prompt) on model accuracy and robustness. **Methods:** We retrospectively analyzed 625 EUS reports for T-staging and 579 for N-staging, which were collected from 663 patients at the Sun Yat-sen University Cancer Center between 2018 and 2020. A 2 × 2 factorial design (Language × Prompt) was employed under a zero-shot setting. The performance of the models was evaluated using accuracy, and the odds ratio (OR) was calculated to quantify the comparative performance advantage between models across different scenarios. **Results:** Performance was evaluated across four scenarios: (1) Chinese with-prompt, (2) Chinese without-prompt, (3) English with-prompt, and (4) English without-prompt. In both T and N-staging tasks, DeepSeek-R1 demonstrated superior overall performance compared to the competitors. For T-staging, the average accuracy was (DeepSeek-R1 vs. GPT-4o vs. Qwen3 vs. Grok-3: 91.4% vs. 84.2% vs. 89.5% vs. 81.3%). For N-staging, the respective average accuracy was 84.2% vs. 65.0% vs. 68.4% vs. 51.9%. Notably, N-staging proved more challenging than T-staging for all models, as indicated by lower accuracy. This superiority was most pronounced in the Chinese without-prompt T-staging scenario, where DeepSeek-R1 achieved significantly higher accuracy than GPT-4o (OR = 7.84, 95% CI [4.62–13.30], *p* < 0.001), Qwen3 (OR = 5.00, 95% CI [2.85–8.79], *p* < 0.001), and Grok-3 (OR = 6.47, 95% CI [4.30–9.74], *p* < 0.001). **Conclusions:** This study validates the feasibility and effectiveness of large language models (LLMs) for automated T/N staging from EUS reports. Our findings confirm that DeepSeek-R1 possesses strong intrinsic reasoning capabilities, achieving the most robust performance across diverse conditions, with the most pronounced advantage observed in the challenging English without-prompt N-staging task. By establishing a standardized, objective benchmark, DeepSeek-R1 mitigates inter-observer variability, and its deployment provides a reliable foundation for guiding precise, individualized treatment planning for esophageal cancer patients.

## 1. Introduction

Large Language Models (LLMs), trained via self-supervised learning on massive, heterogeneous corpora, have acquired profound capabilities in linguistic structuring, world knowledge, and reasoning, demonstrating substantial potential for medical text analysis and zero-shot tasks [1,2,3,4,5,6,7,8,9,10]. Models such as GPT-4o and Qwen have demonstrated efficacy in parsing clinical notes and generating reports. Multiple studies have substantiated the clinical utility of these models in tasks such as automating cancer staging, structuring free-text reports, and supporting clinical decision-making, as summarized in Table 1 based on relevant literature from the past three years. Although they prove effective for routine tasks like structuring electronic health records [11,12,13,14,15], LLMs are no longer confined to language fluency and are beginning to function as engines of deeper cognitive reasoning. This transition is evidenced by the versatile capabilities that LLMs have demonstrated across medical domains [7,16,17,18,19,20,21,22,23,24,25,26,27,28,29,30,31,32]. In particular, the newly emerging DeepSeek-R1 uniquely employs large-scale reinforcement learning to further advance the intrinsic reasoning required for complex report analysis. However, the specific performance of DeepSeek-R1 in analyzing complex medical texts for automated staging remains understudied [23,27], warranting a systematic evaluation to validate its potential as a reliable clinical decision support tool.

In clinical practice, LLM performance is influenced by both the model’s inherent capacities and critical external variables, particularly prompting strategy and language [33,34]. Previous studies have identified key factors affecting LLM performance in medical applications. For instance, Cao et al. reviewed evidence that prompt engineering serves as a core determinant of performance, noting that variations in phrasing can significantly impact output accuracy [35]; Jin et al. observed notable performance degradation with non-English queries such as Chinese [36]; and Zhao et al. emphasized the critical influence of decoding parameters (e.g., temperature) on response consistency [37]. These findings collectively highlight the necessity for defining reliable application conditions for LLMs in clinical tasks (e.g., endoscopic ultrasound report (EUS) staging), including the optimization of prompting strategies and language adaptation.

Endoscopic ultrasound (EUS)-based TNM staging is crucial for managing esophageal cancer. EUS provides a clear visualization of the esophageal wall layers and lymph nodes. However, efficiently extracting vital decision-making data from these complex, unstructured EUS reports remains a significant challenge for endoscopists, who are often encumbered by subjectivity and information overload. Such variability in staging interpretation can compromise the reliability of treatment decisions. Consequently, automating this staging process with LLMs offers clear clinical value by establishing a standardized, objective benchmark that mitigates inter-observer variability and reduces clinician workload. This consistency can help precise, individualized treatment planning and facilitate seamless information transfer for patients across different medical institutions.

Based on the above considerations, we conduct a study to comprehensively benchmarking the performance of advanced reasoning-focused models—DeepSeek-R1, GPT-4o, Qwen3, and Grok-3 for automated EUS staging and to systematically examines the impact of key influencing factors. We primarily aim to assess whether these models can leverage their intrinsic reasoning capabilities to overcome the challenge of variability in clinical interpretation and provide more objective, consistent staging. This could establish a more reliable foundation for guiding individualized precision therapy.

## 2. Materials and Methods

T/N staging was evaluated strictly in accordance with the 8th edition of the Union for International Cancer Control (UICC) TNM classification system for esophageal cancer. The specific definitions for tumor depth (T1a–T4b) and regional lymph node metastasis (N0–N3) utilized in this study—and incorporated into the LLM prompts—are detailed in Supplementary Material (Sections S1 and S2).

### 2.1. Dataset

This study’s data were sourced from the Sun Yat-sen University Cancer Center. We retrospectively collected EUS reports from 663 patients initially diagnosed with esophageal cancer between 2018 and 2020. These original reports were recorded as unstructured free-text narratives rather than semi-structured templates. Consequently, the included reports exhibited significant heterogeneity in reporting styles and terminology. For instance, tumor descriptions varied widely among endoscopists, ranging from concise qualitative summaries (e.g., wall thickening) to detailed assessments of lesion dimensions and echo-texture. To ensure data quality and relevance, reports were screened according to preset criteria. These criteria excluding three categories: (a) cases with missing reports or reports lacking textual content (*n* = 4); (b) cases lacking evaluable staging information either because esophageal stenosis/obstruction precluded endoscope passage (*n* = 19); or because examination termination stemming from patient issues (*n* = 15). Additionally, all reports that met the screening criteria were double-checked by an assessment team. This group, composed of three EUS endoscopists, each with over 10 years of clinical experience, performed a strict review. To mitigate the inherent inter-observer variability and ensure a reliable gold standard, we adopted a majority-voting consensus protocol. Specifically, each report was independently reviewed by the three experts, and the final staging label was determined by the agreement of at least two experts. Cases with persistent disagreement were resolved through group discussion to reach a consensus. Following screening, the final dataset comprised 625 reports containing explicit T-staging information; among these, 579 reports also contained analyzable N-staging information, which was utilized for N-staging analysis. The distributions of T-staging and N-staging are presented in Table 2.

### 2.2. TNM Staging

T/N staging was evaluated strictly in accordance with the 8th edition of the Union for International Cancer Control (UICC) TNM classification system for esophageal cancer. The specific definitions for tumor depth (T1a–T4b) and regional lymph node metastasis (N0–N3) used in this study are summarized in Table 3.

### 2.3. LLM and Testing Process

This study aimed to compare the performance of DeepSeek-R1 against three other advanced LLMs in the automated extraction of esophageal cancer T/N staging information from endoscopic ultrasound (EUS) reports, with a key focus on evaluating their clinical reasoning capabilities and robustness. The evaluated models were DeepSeek-R1, GPT-4o, Qwen3, and Grok-3. Specifically, we utilized DeepSeek-R1 (v.0528, released 28 May 2025), GPT-4o (API version updated 26 March 2025), Qwen3 (v. Qwen3-235B-A22B, released 28 April 2025), and Grok-3 (preliminary version, released 19 February 2025).

The input text for the models consisted of de-identified EUS reports. Prior to analysis, these reports were standardized by removing all patient identifiers and any existing staging conclusions to prevent label leakage. All models were run in a text-only mode, with functionalities such as retrieval-augmented generation (RAG), multimodal input, and code execution disabled to isolate their core text-based reasoning capabilities.

**Figure 1:**
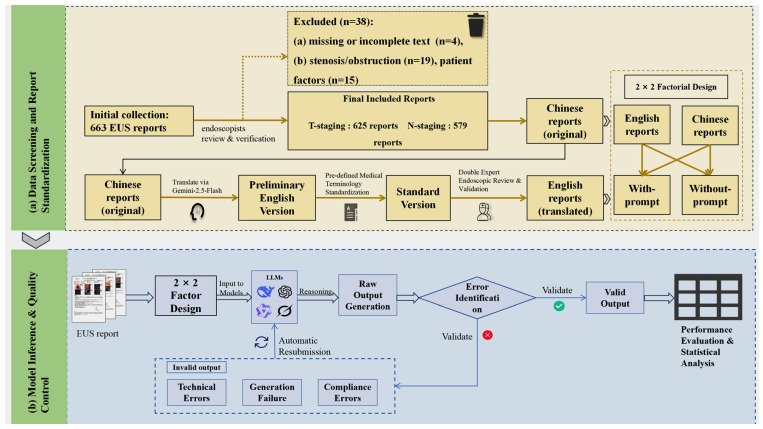
Study workflow. (**a**) Data Screening and Report Standardization: Visualizes the patient inclusion/exclusion process and the rigorous three-stage translation pipeline used to construct the standardized bilingual dataset. (**b**) Model Inference and Quality Control: Details the zero-shot inference protocol and the automated error handling mechanism.

**Figure 2:**
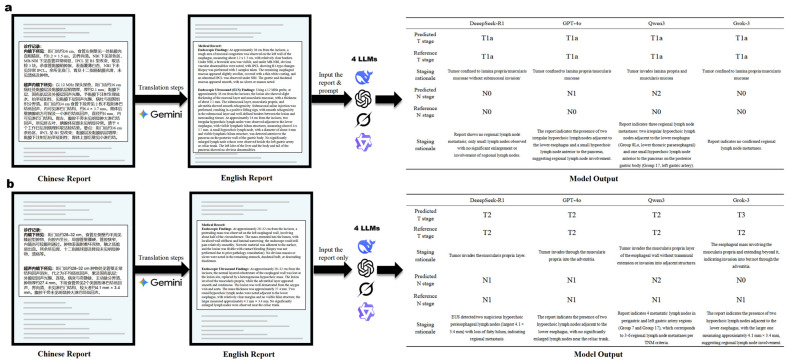
Schematic illustration of model performance under with-/without-prompt conditions. (**a**) English with-prompt: A de-identified Chinese EUS report is translated into an English version (Gemini) and submitted to four LLMs with a standardized prompt. Results are presented as predicted and reference stages, together with a staging rationale. (**b**) English without-prompt: A de-identified Chinese EUS report is translated into English (Gemini) and submitted to the same four LLMs as report text in without-prompt condition. Results are presented as predicted and reference stages, together with a staging rationale.

This study used Python (version 3.13) and the pandas (version 2.2.3), dashscope (version 1.23.2), and OpenAI libraries (version 1.78.0), adopting a zero-shot learning approach. Custom Python scripts communicated with the LLMs via their respective Application Programming Interfaces (APIs), with the temperature parameter set to zero to ensure output determinism. To ensure output quality, a standardized control protocol was implemented. As detailed in Table 4, errors were classified as Technical Errors or Model Output Errors [38,39,40], the latter of which was subdivided into Invalid Output and Non-compliant Output. The protocol for all error types was to resubmit the original EUS report and the corresponding prompt until a syntactically valid response was obtained. A response was considered syntactically valid if it adhered to the predefined “Staging/Reason” format and provided a result within the predefined staging categories. For the final performance analysis, only the first syntactically valid response from each model for each report was used. This approach ensured a consistent basis for comparing the models’ initial reasoning capabilities. The comprehensive study workflow is illustrated in Figure 1, and two illustrative examples of the model input–output process under the English with-prompt and English without-prompt conditions are presented in Figure 2.

### 2.4. Influencing Factors

#### 2.4.1. Prompt Strategy

We implemented two contrasting strategies to investigate the influence of prompting. This design aimed to distinguish between instruction-following capabilities and intrinsic medical reasoning. In the with-prompt condition, detailed UICC staging criteria, and strict formatting rules were incorporated to evaluate the models’ adherence to explicit external instructions. In the without-prompt condition, these auxiliary definitions were omitted to assess the models’ ability to perform staging tasks relying solely on pre-trained internal knowledge and reasoning.

Specifically: In the with-prompt condition, a composite instruction was submitted that: (a) set the model’s role as a “Professional Digestive Endoscopist”; (b) provided detailed definitions of the UICC 8th edition TNM staging criteria for T or N staging; and (c) required adherence to a predefined strict output template to return the result in the “Staging/reason” format. This multi-step instruction ensured the standardization and parsability of the output. In the without-prompt condition, the model only received the EUS report text and was merely instructed to play the role of a “Professional Digestive Endoscopist.” The LLM was then asked to directly output the T or N staging result and reason, without additional staging definitions. To ensure reproducibility, the full text of the Chinese and English instructions for both the “with/without-prompt” conditions across the T and N staging tasks are available in the Supplementary Material (Sections S1 and S2).

#### 2.4.2. Language Environment

To rigorously evaluate the models’ cross-lingual robustness and simulate a global deployment scenario, the study utilized a bilingual dataset comprising original Chinese reports and their English translations. To minimize semantic drift during this process, a rigorous three-stage translation-validation protocol was implemented: (1) generating an initial English draft using the Gemini-2.5-Flash model, chosen for its advanced multilingual capabilities; (2) standardizing key anatomical and pathological terms (e.g., ‘muscularis propria’, ‘adventitia’) were mechanically standardized against a pre-defined Medical Terminology Library derived from the UICC 8th edition guidelines to ensure strict terminological consistency; and (3) conducting a final audit by two EUS endoscopists, each with over 10 years of clinical experience. This audit ensured high consistency between the translated content and the core medical semantics of the original reports. This multi-step process ensured maximal consistency in medical terminology, core content, and structure between the original Chinese and translated English reports, thereby minimizing potential interference from language differences in the subsequent model evaluation.

### 2.5. Statistical Analysis

This study employed a 2 × 2 factorial design, resulting in four independent analysis scenarios: (1) Chinese with-prompt, (2) Chinese without-prompt, (3) English with-prompt, and (4) English without-prompt. Model performance was evaluated using the following metrics. First, we used accuracy as the fundamental measure of correctness. Second, we employed the QWK to measure agreement [41,42]; given the ordinal nature of TNM staging, this metric is particularly important as it penalizes larger discrepancies more heavily. Then, to assess whether these differences existed among the models and were statistically significant, we employed Cochran’s Q test, where a larger Q-statistic indicates greater disagreement among model accuracies. We subsequently used pairwise McNemar tests to pinpoint specific performance gaps between model pairs. For each comparison, we reported the odds ratio (OR) with its 95% confidence interval (CI) to quantify the effect size and the *p*-value. The two-sided *p* < 0.05 was considered statistically significant (* *p* < 0.05, ** *p* < 0.01, *** *p* < 0.001). All statistical analyses were performed using R software (version 4.4.3). To ensure a comprehensive and unbiased evaluation, we additionally calculated Precision, Macro-Recall, and Macro-F1 scores [16,43]. These multi-dimensional metrics are detailed in Supplementary Tables S3 and S4.

## 3. Results

### 3.1. Characteristics of the EUS Reports

This study ultimately included 625 EUS reports for T-stage analysis and 579 for N-stage analysis. As detailed in Table 2, the baseline characteristics of the study cohort indicated a mean patient age of 61.6 years (standard deviation ± 7.8 years). The majority of patients were male (502 cases, 80.3%). Pathologically, the T-staging was predominantly T3, accounting for 285 cases (45.6%), while the N-staging was most frequently N2, totaling 201 cases (34.7%).

### 3.2. Overall Performance

**Figure 3:**
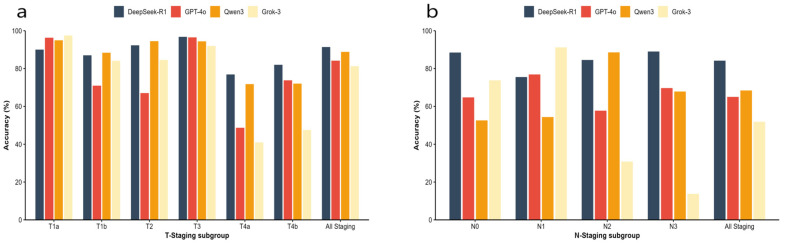
The comparison of average accuracy among the four models across different T and N staging. (**a**) Average accuracy across T-staging subgroups (T1a–T4b) and overall average accuracy for T-staging. (**b**) Average accuracy across N-staging subgroups (N0–N3) and overall average accuracy for N-staging.

The comparison of average accuracy among the four models across different T and N staging subgroups is presented in Table 5 and visually summarized in Figure 3. Detailed results for each stage and scenario are provided in Supplementary Material (Tables S1–S4). In the T-staging task, DeepSeek-R1 demonstrated the highest overall average accuracy of 91.4%, outperforming Qwen3 (88.8%), GPT-4o (84.2%), and Grok-3 (81.3%). While all models achieved respectable accuracy in the frequently occurring T3 subgroup, DeepSeek-R1 maintained more stable performance across other T-stage subgroups compared to its competitors. The N-staging task proved significantly more challenging than T-staging for all models. Nevertheless, DeepSeek-R1 retained a substantial lead with an overall average accuracy of 84.2%, significantly surpassing Qwen3 (68.4%), GPT-4o (65.0%), and Grok-3 (51.9%). Regarding subgroup performance, DeepSeek-R1 demonstrated exceptional stability, achieving its highest accuracy in the N3 subgroup (88.5%), whereas Grok-3 exhibited severe performance degradation in the same category (13.8%). Furthermore, a comprehensive evaluation encompassing additional multi-dimensional metrics, including Quadratic Weighted Kappa (QWK), Macro-Recall, and Macro-F1 score, were performed to assess model reliability across all classification stages. The detailed results of these metrics, consistently demonstrating DeepSeek-R1′s superior performance across both T and N staging, are provided in Supplementary Material (Tables S3 and S4).

### 3.3. Analysis of Influencing Factors

**Figure 4:**
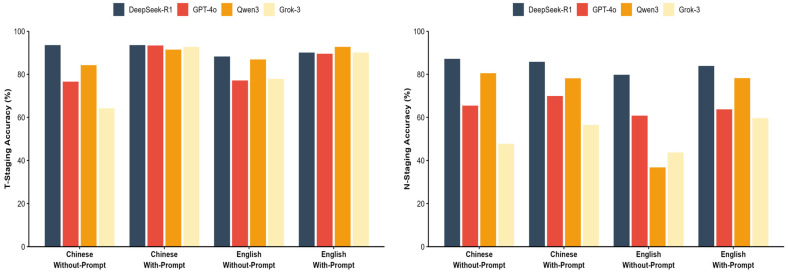
Impact of prompt strategy and language. Visual comparison of model accuracy across Chinese/English and with/without-prompt conditions.

The impact of prompting strategies and language environments on model performance was evaluated using a 2 × 2 factorial design, with detailed accuracy data presented in Table 6 and Figure 4. Specifically, in the with-prompt conditions, performance variation was minimal, all models achieved accuracy exceeding 91%, with Cochran’s Q test confirming no significant difference (Q = 4.97, *p* = 0.174). In without-prompt scenarios, a notable heterogeneity in model capabilities emerged. DeepSeek-R1 maintained superior performance (e.g., 93.6% for Chinese T-staging) while competitors exhibited varying degrees of decline. This performance disparity was statistically significant, peaking in the Chinese N-staging scenario (Cochran’s Q = 271.23, *p* < 0.001).

Regarding the impact of language, while most models showed decreased accuracy when transitioning from Chinese to English contexts, DeepSeek-R1 demonstrated cross-lingual stability. In the challenging English without-prompt N-staging scenario, DeepSeek-R1 retained an accuracy of 79.8%. In contrast, Qwen3 showed a marked reduction in performance, with accuracy dropping to 36.8%, indicating a susceptibility to language and prompt variations in this specific task.

### 3.4. Pairwise McNemar Test Comparison Across Scenarios

**Figure 5:**
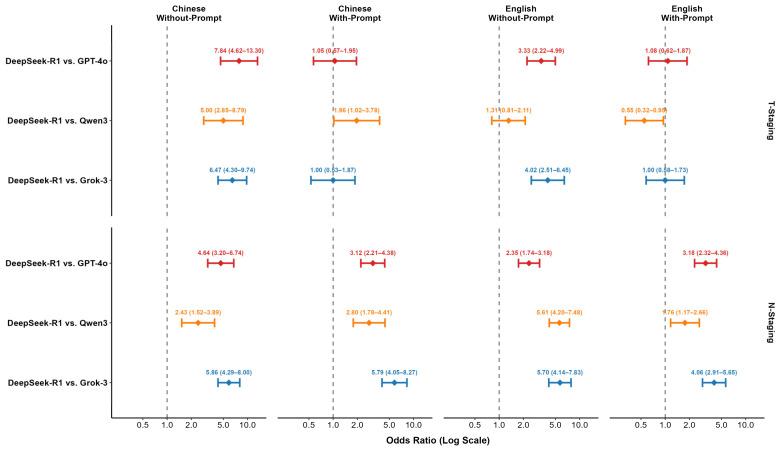
Forest plot of Odds Ratios (OR) and 95% CIs comparing DeepSeek-R1 against competing models.

Pairwise McNemar tests were conducted to rigorously quantify performance differences, with DeepSeek-R1 serving as the reference baseline. The comparative results, expressed as Odds Ratios (OR), are presented in Table 7 and visualized in the forest plot in Figure 5. In the with-prompt conditions, performance differences were generally not statistically significant for T-staging. For the N-staging task, DeepSeek-R1 showed a higher accuracy than all competitors, reaching statistical significance in the Chinese condition against GPT-4o (OR = 3.12, 95% CI [2.21–4.38], *p* < 0.001), Qwen3 (OR = 2.80, 95% CI [1.78–4.41], *p* < 0.001), and Grok-3 (OR = 5.79, 95% CI [4.05–8.27], *p* < 0.001), as well as against all three competitors in the English condition.

In the without-prompt conditions, performance gaps became more pronounced. For T-staging, DeepSeek-R1′s performance was significantly superior to its competitors in several scenarios, notably against GPT-4o (OR = 7.84, 95% CI [4.62–13.30], *p* < 0.001) and Grok-3 (OR = 6.47, 95% CI [4.30–9.74], *p* < 0.001) in the Chinese condition. This trend was equally evident in the N-staging task, where DeepSeek-R1 significantly outperformed all three competing models across all without-prompt scenarios. For instance, in the Chinese without-prompt N-staging condition, DeepSeek-R1′s superiority was substantial against GPT-4o (OR = 4.64, 95% CI [3.20–6.74], *p* < 0.001), Qwen3 (OR = 2.43, 95% CI [1.52–3.89], *p* < 0.001), and Grok-3 (OR = 5.86, 95% CI [4.29–8.00], *p* < 0.001). A complementary evaluation using Quadratic Weighted Kappa (QWK), Macro-F1, and Macro-Recall further validated this trend. DeepSeek-R1 maintained high agreement with the gold standard (QWK ≥ 0.84) and achieved high Macro-Recall scores across all conditions, reflecting balanced performance even in subgroups with smaller sample sizes. Conversely, competitors exhibited marked declines in these metrics, particularly in the without-prompt scenarios (Supplementary Table S3).

## 4. Discussion

This study systematically evaluated the performance of four advanced reasoning models on T/N staging from esophageal cancer EUS reports. More importantly, we innovatively employed a 2 × 2 factorial design to explore how crucial external variables—language settings and prompting strategies—affect model performance. Our findings reveal that DeepSeek-R1 consistently outperformed its competitors because it not only demonstrated superior accuracy but also maintained strong robustness across changing conditions, especially in challenging without-prompt scenarios. This combination of high accuracy and robustness suggests a more advanced intrinsic reasoning capability, highlighting its potential as a reliable clinical decision-support tool. By enhancing staging consistency and objectivity, the model can support endoscopists in T/N staging for patients with esophageal cancer, thereby establishing a more reliable foundation for guiding individualized precision therapy.

The application of LLMs to extract structured information from unstructured clinical texts has been validated across multiple oncology document types [6,8]. In lung and breast cancer settings, models such as the GPT series can extract TNM elements from radiology, pathology, and ultrasound reports to generate structured outputs [3,7,21]. These approaches have been extended to other cancer types and general clinical note analysis [32,44]. EUS for esophageal cancer provides a crucial basis for preoperative local staging, yet the interpretation of these complex EUS reports often varies significantly among endoscopists, leading to discrepancies in staging. This limitation highlights the urgent need for automation to enhance consistency in this domain [45,46]. This study explores the clinical value of applying various LLMs to EUS report analysis. We envision embedding DeepSeek-R1 into the electronic medical record or endoscopic reporting system to provide real-time decision support. In this workflow, once the endoscopist completes the free-text description, the LLM would instantly analyze the narrative to infer the T/N stage. The system would not automatically overwrite the endoscopist’s conclusion but instead flag potential discrepancies for review, thereby ensuring standardization without disrupting decision-making authority. Ultimately, this may facilitate the development of a stable, objective, and highly accurate LLM tool capable of effectively assisting clinicians in reducing diagnostic variability and establishing a more reliable foundation for guiding individualized precision therapy.

Furthermore, to systematically evaluate the key factors influencing model performance, this study implemented three specific methodological improvements: we conducted a parallel evaluation of DeepSeek-R1—optimized via large-scale reinforcement learning—against general models aligned through supervised fine-tuning to compare different training paradigms for clinical reasoning tasks [32,47]; we introduced a without-prompt condition to distinguish instruction-following from intrinsic reasoning, thereby addressing prior reliance on prompt engineering; and we employed a Language × Prompt 2 × 2 design to examine their interaction and cross-lingual transfer, using metrics such as accuracy and QWK to evaluate performance [33]. Furthermore, by strictly adhering to a zero-shot setup, we eliminated the selection bias inherent in few-shot example curation and avoided the computational overhead of fine-tuning. This design choice rigorously benchmarks the models’ intrinsic reasoning for direct, resource-efficient clinical deployment. This methodological positioning establishes a clear framework for subsequent experiments and result interpretation.

In our study, all models demonstrated high performance levels under ideal conditions with explicit instructions, achieving > 90% accuracy in T-staging tasks and indicating strong baseline capabilities. However, under the more challenging without-prompt scenarios, model performance diverged substantially. This divergence was particularly evident for N-staging. For instance, in the English without-prompt condition, DeepSeek-R1 achieved a significantly higher accuracy of 79.8%, compared to 60.8% for GPT-4o, 43.7% for Grok-3, and 36.8% for Qwen3. DeepSeek-R1 exhibited exceptional accuracy and robustness with minimal accuracy fluctuations, while the other models showed varying degrees of performance degradation. This divergence highlights a critical distinction in model capabilities: some models are highly dependent on prompting, whereas others possess superior intrinsic reasoning. In a real-world clinical setting with variable reporting styles, a model with lower dependence on prompts may be more reliable and valuable as a decision-support tool.

In addition to accuracy, the consistency of a model’s staging results is crucial for evaluating its clinical value. Reliable staging is the cornerstone for formulating subsequent treatment strategies. For esophageal cancer, precise T-staging is crucial for guiding therapeutic approaches from endoscopic resection to surgery or neoadjuvant therapy, while N-staging is a key determinant of systemic treatment decisions. However, this critical process is often challenged by the variability in the clinical interpretation of EUS reports. Therefore, to evaluate the clinical impact of staging errors, we analyzed the quadratic weighted kappa (QWK), a metric that penalizes severe, cross-stage misclassifications more heavily. Although all models demonstrated high agreement with prompts, their removal revealed marked differences in consistency. DeepSeek-R1 maintained excellent agreement even in the most challenging English without-prompt N-staging task (QWK = 0.84), whereas Qwen3′s agreement collapsed (QWK = 0.02) and Grok-3′s also declined significantly (QWK = 0.52). The near-zero QWK value for Qwen3 signifies a performance collapse in this scenario; as detailed in the confusion matrix (Supplementary Material, Section S4), the model incorrectly classified 91.8% of N0 cases, 89.8% of N1 cases, and 92.7% of N3 cases as N2. This finding indicates that DeepSeek-R1′s predictions demonstrate not only superior accuracy but also a more consistent and safer alignment with the gold standard. In clinical practice, this consistency has the potential to standardize interpretations among endoscopists, thereby contributing to a reduction in staging-related risks.

Our data reveal several key performance characteristics of DeepSeek-R1 that align with crucial clinical needs. First, a cross-level staging error can lead to drastically different treatment plans. The contrast in error patterns is quantified by our analysis of the confusion matrices (Supplementary Material, Section S3). For the Chinese without-prompt N-staging task, DeepSeek-R1 misclassified N1 cases almost entirely to adjacent stages N0 (8.2%) and N2 (12.9%), with a minimal rate (1.4%) of non-adjacent (N3) errors. This pattern stands in stark contrast to the egregious cross-stage errors seen in other models. Grok-3, for example, misclassified 30.3% of N3 cases as the distant N1 stage under identical conditions (Supplementary Material, Figures S1–S4). Clinically, such distant misclassifications carry profound risks: erroneously staging an N0 patient as N2 could trigger unnecessary surgical and endoscopic procedures, that may lead to major treatment escalation, while confusing N1 with N3 might lead to inaccurate prognostication and therapeutic mismatch, which carries risk of overtreatment. In contrast, DeepSeek-R1′s errors were predominantly confined to adjacent stages, a conservative pattern that minimizes the likelihood of radical shifts in therapeutic strategy. This safer error profile further underscores its potential as a reliable clinical decision-support tool. This demonstrates DeepSeek-R1′s clinically safer “conservative error” pattern, mitigating the risk of severely inappropriate treatment decisions. Second, to address the challenge of varied report styles in clinical practice, a model must possess strong intrinsic reasoning. DeepSeek-R1 showcased this by maintaining a high accuracy of 93.6% in the Chinese T-staging task even without prompts. In contrast, competitors’ performance dropped under the same conditions, with Grok-3′s accuracy falling to 64.2%. This low dependence on prompts makes it a more practical and adaptable tool. Third, for broader applications like multi-center trials, cross-lingual robustness is essential. In the challenging English without-prompt N-staging task, DeepSeek-R1 achieved a high accuracy of 79.8%, outperforming all competitors: GPT-4o (60.8%), Grok-3 (43.7%), and Qwen3 (36.8%). This supports its potential for reliable deployment across diverse linguistic environments.

This study has several limitations. First, the data were obtained from a single center, and reporting styles may exhibit institution-specific characteristics, limiting the generalizability of our conclusions; future validation through multi-center studies is warranted. Second, although the selected models are architecturally representative, they do not cover the full spectrum of parameter scales; expanding the model range is needed to establish a more comprehensive evaluation framework. Third, the experiments used only a zero-shot setting and did not explore potential gains from strategies such as few-shot learning. Fourth, while our strict three-stage translation protocol ensured semantic equivalence, we acknowledge that the intrinsic syntactic structures and phrasing of English may impose subtle constraints on the models’ interpretation of the text. These linguistic nuances, distinct from the original Chinese context, could partially contribute to the observed performance variations across languages. These limitations point to valuable directions for future research, including validating clinical applicability in multi-center environments and systematically assessing the effects of different learning strategies.

## 5. Conclusions

This study confirms that the practical utility of LLMs in clinical settings depends on their robust and consistent performance across diverse conditions. DeepSeek-R1 exemplifies this principle through its safer error profiles and low dependence on prompts, establishing a benchmark for a clinically trustworthy LLM. Future work should prioritize validating these reliability-focused attributes in multi-center studies to accelerate real-world clinical adoption. In conclusion, DeepSeek-R1′s superior accuracy, robustness, and low dependence on prompts make it a promising clinical tool for enhancing clinical decision support. It shows significant potential to provide reliable T/N staging support for endoscopists and aid in guiding personalized treatment decisions for patients with esophageal cancer. Future studies are warranted to prioritize multi-center validation to accelerate its clinical adoption.

## Figures and Tables

**Table 1:** Summary of recent studies utilizing Large Language Models (LLMs) for oncology tasks and structured information extraction.

Author	Cancer Type and Modality	LLMs Investigated	Task and Focus	Statistical Metrics	Statistical Tests	Limitations/Significance
Choi et al. (2023) [3]	Breast Cancer (Pathology and Ultrasound)	GPT-3.5-turbo	Extracting clinical factors, focusing on prompt development efficiency (time/cost).	Accuracy, Time consumption, Cost analysis	Descriptive comparison (Manual vs. LLM)	Validates the efficiency of LLM prompting for basic extraction but lacks analysis of complex reasoning or logic consistency.
Matsuo et al. (2024) [7]	Lung Cancer (Chest CT)	GPT-3.5-turbo	Multilingual TNM classification, evaluating the impact of providing definitions prompts	Accuracy	Generalized Linear Mixed Model (GLMM), Odds Ratio (OR)	Validates that external definition injection improves accuracy, but performance remains lower in non-English contexts compared to English.
Bhayana et al. (2024) [16]	Pancreatic Cancer (CT Reports)	GPT-4, GPT-3.5	Resectability Categorization, focus on prompt logic	F1-score, Precision, Recall, Accuracy	McNemar’s Test, Wilcoxon signed-rank test	Validates that Chain-of-Thought prompting improves categorization logic.
Lee et al. (2024) [19]	Lung Cancer (Multimodality Radiology)	GPT-3.5, GPT-4, GPT-4o	Automated TNM staging, benchmarking LLM performance against human radiologists of varying experience	Overall Staging Accuracy, Error Rate	McNemar’s Test	Establishes that LLMs can match physicians in TNM staging.
Nakamura et al. (2023) [21]	Lung Cancer (CT Reports)	GPT-4, GPT-3.5 Turbo	Feasibility of automated staging, identifying failures in numerical reasoning	Accuracy	Descriptive analysis of error types	Validates staging feasibility but exposes frequent errors in handling numerical thresholds and anatomical details due to insufficient reasoning capabilities.
He et al. (2025) [27]	Esophageal Cancer (QA Tasks)	DeepSeek-R1, Gemini 2.5, ChatGPT-5, Grok-4	Evaluating accuracy and completeness of esophageal cancer Q&A.	Accuracy score, Completeness score (Likert scale)	Friedman test, Wilcoxon signed-rank, Bonferroni correction	Highlights trade-offs: Gemini excelled in accuracy while ChatGPT led in completeness; supports a tiered model selection strategy for clinical consulting.
Ishida et al. (2025) [28]	Gynecologic Cancer (Pathology Reports)	Gemini 1.5 Pro, Qwen2.5-72B	Automated TNM staging via zero-shot prompting without model fine-tuning.	Accuracy, Error Rate	Comparison with manual registry error rates	Validates that simple prompting outperforms manual entry (>99% accuracy).
Kim et al. (2025) [29]	Colorectal Cancer (Imaging Reports)	GPT-4	Clinical Staging Extraction: Extracting TNM stages and lesion locations from unstructured reports using specific prompts.	Accuracy	Chi-square test	Demonstrates high accuracy in English-only contexts; notably, LLMs outperformed human data managers in extracting precise lesion locations.
Papale et al. (2025) [30]	Pancreatic Cysts (Radiology Reports)	GPT-4	Comparing “Open-Prompt” vs. “Entity-Extraction” for risk classification.	F1-score, Precision, Recall	95% Confidence Intervals (CI)	Validates the superiority of “Entity-Extraction” prompting over generic prompts in identifying high-risk cysts.
Yao et al. (2025) [31]	Esophageal Cancer (Radiology Reports)	INF-72B, Qwen2.5-72B, LLaMA3.1	Preoperative Staging: Comparing prompting strategies (Zero-shot, CoT, Interpretable Reasoning) vs. clinicians.	Accuracy, F1-score	McNemar test, Pearson chi-square	Validates that “Interpretable Reasoning” prompting significantly enhances LLM performance, matching or surpassing clinicians in staging accuracy.
Luo et al. (2025) [32]	Prostate Cancer (Radiotherapy)	DeepSeek-R1, GPT-4o	Bilingual QA for patient education and clinical consultation	Physician Satisfaction (Likert Scale)	Wilcoxon signed-rank test, Student’s *t*-test, Mann–Whitney U-test	Validates DeepSeek’s superior performance in Chinese contexts but focuses on simple QA retrieval rather than complex staging logic.
Our Study	Esophageal Cancer (EUS Reports)	DeepSeek-R1, GPT-4o, Qwen3, Grok-3	Zero-shot T/N staging (Focus on Intrinsic Reasoning Robustness)	Accuracy, Quadratic Weighted Kappa (QWK)	Cochran’s Q test, McNemar’s Test, Odds Ratio (OR)	Validates DeepSeek-R1′s intrinsic reasoning ensures robustness in unprompted and cross-lingual staging.

**Table 2:** Baseline characteristics and T/N staging distribution of the study sample.

Characteristic	Value
**Age (years), mean ± SD**	61.6 ± 7.8
**Sex, *n* (%)**	
Male	502 (80.3)
Female	123 (19.7)
**Endoscopic T/N staging**	
T stage (*n* = 625), *n* (%)	
T1a	80 (12.8)
T1b	69 (11.0)
T2	91 (14.6)
T3	285 (45.6)
T4a	39 (6.2)
T4b	61 (9.8)
N stage (*n* = 579), *n* (%)	
N0	122 (21.1)
N1	147 (25.4)
N2	201 (34.7)
N3	109 (18.8)

SD, standard deviation; *n*, number of patients; T, Tumor; N, Node.

**Table 3:** Definitions for T and N categories in esophageal cancer EUS staging.

Category	Definition
**T: Primary Tumor**	
T1a	Tumor invades the lamina propria, muscularis mucosae, or submucosa
T1b	Tumor invades the lamina propria or muscularis mucosae
T2	Tumor invades the submucosa
T3	Tumor invades the muscularis propria
T4a	Tumor invades the adventitia
T4b	Tumor invades neighboring structures
**N: Regional** **Lymph Nodes**	
N0	No metastasis in the regional lymph nodes
N1	Metastasis in 1–2 regional lymph nodes
N2	Metastasis in 3–6 regional lymph nodes
N3	Metastasis in 7 or more regional lymph nodes

**Table 4:** Error classification and handling protocol for LLM outputs.

Error Category	Specific Error Type	Description	Handling Method
Technical Errors	The network is busy, or call failure	Failed to obtain any response from the model due to network timeout, server issues, or configuration errors.	Resubmit the original report and prompt, with a maximum of 5 retries. If the failure persists, it is recorded as a technical fault.
Model Output Errors	A. Generation Failure		
	Return blank results	The model failed to generate any valid or relevant text content.	Resubmit the original report and prompt repeatedly until a non-blank, properly formatted response is obtained.
	Refusals to answer or irrelevant dialogue	The model returned a refusal to process the request or generated dialogue text irrelevant to the task.	Resubmit the original report and prompt repeatedly until a task-relevant, properly formatted response is obtained.
	B. Format/Compliance Errors		
	Unparsable text	The model’s response did not adhere to the predefined “Stage/Reason” output format.	Resubmit the original report and prompt repeatedly until a response that can be programmatically parsed is obtained.
	Non-compliant staging results	The model returned a staging result outside the study’s predefined categories (e.g., T2a, N4) or an ambiguous stage (e.g., Tx).	Resubmit the original report and prompt repeatedly until a response that uses a compliant staging category is obtained.
Error Category	Specific Error Type	Description	Handling Method
Technical Errors	The network is busy, or call failure	Failed to obtain any response from the model due to network timeout, server issues, or configuration errors.	Resubmit the original report and prompt, with a maximum of 5 retries. If the failure persists, it is recorded as a technical fault.
Model Output Errors	A. Generation Failure		
	Return blank results	The model failed to generate any valid or relevant text content.	Resubmit the original report and prompt repeatedly until a non-blank, properly formatted response is obtained.
	Refusals to answer or irrelevant dialogue	The model returned a refusal to process the request or generated dialogue text irrelevant to the task.	Resubmit the original report and prompt repeatedly until a task-relevant, properly formatted response is obtained.
	B. Format/Compliance Errors		
	Unparsable text	The model’s response did not adhere to the predefined “Stage/Reason” output format.	Resubmit the original report and prompt repeatedly until a response that can be programmatically parsed is obtained.
	Non-compliant staging results	The model returned a staging result outside the study’s predefined categories (e.g., T2a, N4) or an ambiguous stage (e.g., Tx).	Resubmit the original report and prompt repeatedly until a response that uses a compliant staging category is obtained.

**Table 5:** Comparative average accuracy of four models across T and N staging subgroups.

Staging Category	Subgroup	DeepSeek-R1	GPT-4o	Qwen3	Grok-3
**T-Staging**	Overall Accuracy	91.4%	84.2%	88.8%	81.3%
	T1a (*n* = 80)	90.3%	96.6%	85.3%	97.5%
	T1b (*n* = 69)	86.2%	71.0%	88.0%	83.7%
	T2 (*n* = 91)	92.6%	66.5%	94.2%	84.1%
	T3 (*n* = 285)	96.8%	96.6%	94.2%	91.8%
	T4a (*n* = 39)	76.3%	48.7%	70.5%	39.7%
	T4b (*n* = 61)	81.1%	73.8%	72.1%	47.5%
**N-Staging**	Overall Accuracy	84.2%	65.0%	68.4%	51.9%
	N0 (*n* = 122)	88.1%	64.3%	52.0%	73.6%
	N1 (*n* = 147)	75.5%	76.7%	54.1%	91.0%
	N2 (*n* = 201)	84.3%	57.7%	88.6%	30.6%
	N3 (*n* = 109)	88.5%	69.5%	67.7%	13.8%

**Table 6:** Comparative accuracy and statistical heterogeneity of four models across different languages and prompting scenarios.

Staging Task	Scenario	DeepSeek-R1	GPT-4o	Qwen3	Grok-3	All Models Avg.	Cochran’s Q	*p*-Value
T-Staging (*n* = 625)	Chinese Without-Prompt	93.6% (585/625)	76.6% (479/625)	84.3% (527/625)	64.2% (401/625)	79.7% (498/625)	226.77	<0.001 ***
	Chinese With-Prompt	93.6% (585/625)	93.4% (584/625)	91.5% (572/625)	92.8% (580/625)	92.8% (580/625)	4.97	0.17
	English Without-Prompt	88.3% (552/625)	77.1% (482/625)	86.9% (543/625)	77.9% (487/625)	82.6% (516/625)	68.49	<0.001 ***
	English With-Prompt	90.1% (563/625)	89.6% (560/625)	92.8% (579/625)	90.1% (563/625)	90.7% (567/625)	10.09	0.018 *
N-Staging (*n* = 579)	Chinese Without-Prompt	87.2% (505/579)	65.4% (379/579)	80.5% (466/579)	47.7% (276/579)	70.2% (406/579)	271.23	<0.001 ***
	Chinese With-Prompt	85.8% (497/579)	69.9% (405/579)	78.1% (452/579)	56.5% (327/579)	72.6% (420/579)	178.56	<0.001 ***
	English Without-Prompt	79.8% (462/579)	60.8% (352/579)	36.8% (213/579)	43.7% (253/579)	55.3% (320/579)	239.01	<0.001 ***
	English With-Prompt	83.9% (486/579)	63.7% (369/579)	78.2% (453/579)	59.6% (345/579)	71.4% (413/579)	143.37	<0.001 ***

* *p* < 0.05, *** *p* < 0.001.

**Table 7:** Pairwise comparative advantage of DeepSeek-R1 against competitors based on McNemar’s tests and Odds Ratios (OR).

Staging Category	Comparison Pair (vs. DeepSeek-R1)	Chinese Without-Prompt	Chinese With-Prompt	English Without-Prompt	English With-Prompt
T-Staging	vs. GPT-4o	7.84 (4.62–13.30), *p* < 0.001 ***	1.05 (0.57–1.95), *p* > 0.99	3.33 (2.22–4.99), *p* < 0.001 ***	1.08 (0.62–1.87), *p* > 0.99
	vs. Qwen3	5.00 (2.85–8.79), *p* < 0.001 ***	1.96 (1.02–3.78), *p* = 0.16	1.31 (0.81–2.11), *p* = 0.985	0.55 (0.32–0.95), *p* = 0.121
	vs. Grok-3	6.47 (4.30–9.74), *p* < 0.001 ***	1.00 (0.53–1.87), *p* > 0.99	4.02 (2.51–6.45), *p* < 0.001 ***	1.00 (0.58–1.73), *p* > 0.99
N-Staging	vs. GPT-4o	4.64 (3.20–6.74), *p* < 0.001 ***	3.12 (2.21–4.38), *p* < 0.001 ***	2.35 (1.74–3.18), *p* < 0.001 ***	3.18 (2.32–4.36), *p* < 0.001 ***
	vs. Qwen3	2.43 (1.52–3.89), *p* < 0.001 ***	2.80 (1.78–4.41), *p* < 0.001 ***	5.61 (4.20–7.48), *p* < 0.001 ***	1.76 (1.17–2.66), *p* = 0.024 *
	vs. Grok-3	5.86 (4.29–8.00), *p* < 0.001 ***	5.79 (4.05–8.27), *p* < 0.001 ***	5.70 (4.14–7.83), *p* < 0.001 ***	4.06 (2.91–5.65), *p* < 0.001 ***

OR, odds ratio; CI, confidence interval; OR > 1 indicates DeepSeek-R1 outperformed the competitor. * *p* < 0.05, *** *p* < 0.001.

## Data Availability

The data presented in this study are not publicly available due to ethical and privacy restrictions. Data are available from the corresponding author on reasonable request.

## Supplementary Materials

The following supporting information can be downloaded at: https://www.mdpi.com/article/10.3390/diagnostics16020215/s1, Section S1: Prompt Formulations for T-Staging Task; Section S2: Prompt Formulations for N-Staging Task; Section S3: Per-class T/N-Staging Accuracy by Model and Scenario, including Tables S1–S4; Section S4: Confusion Matrices, including Figures S1–S4. Table S1: Per-class T-staging accuracy by model across Language × Prompt scenarios ; Table S2: Per-class N-staging accuracy by model across Language × Prompt scenarios; Table S3: Comprehensive multi-dimensional performance metrics for T-staging by model and scenario; Table S4: Comprehensive multi-dimensional performance metrics for N-staging by model and scenario. Figure S1: DeepSeek-R1 Confusion Matrices; Figure S2: GPT-4o Confusion Matrices; FigureS3: Grok-3 Confusion Matrices; Figure S4: Qwen3 Confusion Matrices.

## Abbreviations

The following abbreviations are used in this manuscript:

API	Application Programming Interface
EUS	Endoscopic Ultrasound
QWK	Quadratic weighted Kappa
LLM(s)	Large Language Model(s)
MIT	Massachusetts Institute of Technology
MoE	Mixture-of-Experts
OR	Odds Ratio
R	The R statistical computing environment
R/L	Right/Left
RL	Reinforcement Learning
SD	Standard Deviation
SFT	Supervised Fine-Tuning
TNM	Tumor–Node–Metastasis
U/M/Lo	Upper/Middle/Lower
UICC	Union for International Cancer Control
RAG	retrieval–augmented generation
95%CI	95% confidence intervals
