# Supplementary material for: Automated Tumor and Node Staging from Esophageal Cancer Endoscopic Ultrasound Reports: A Benchmark of Advanced Reasoning Models with Prompt Engineering and Cross-Lingual Evaluation
Source: Diagnostics (Basel). 2026 Jan 9;16(2):215. doi: 10.3390/diagnostics16020215 (PMC12839693; doi:10.3390/diagnostics16020215)
Supplement: Supplementary file 1 [file diagnostics-16-00215-s001.zip › diagnostics-3962112-supplementary.pdf]

Supplementary Material

Supplementary Section S1: Prompt Formulations for T-Staging Task

S1.1

Presented below is the full designed prompt provided to the LLMs for T-staging of esophageal cancer endoscopic ultrasound (EUS) reports in this study. This prompt was designed to ensure accurate and consistent staging performance through explicit role specification, detailed task definition, and stringent output formatting requirements.

S1.1.1

Complete Prompt Instruction in Chinese:

"你是一位专业的消化内镜医师。
请根据下面提供的食管癌T分期标准（依据UICC第8版），仔细阅读并分析EUS报告，然后给出最可能的T分期。

T分期标准定义:
T1a: 肿瘤侵犯黏膜固有层或黏膜肌层
T1b: 肿瘤侵犯黏膜下层（未达固有肌层）
T2: 肿瘤侵犯食管固有肌层（肌层），但未突破外膜
T3: 肿瘤穿透肌层，侵犯食管外膜（纤维膜），但未累及邻近结构。
T4a: 侵犯可切除的邻近结构（如胸膜、心包、膈肌、奇静脉、腹膜等）。
T4b: 侵犯无法切除的邻近结构（如主动脉、椎体、气管、支气管、肺等大血管或重要器官）。

输出格式要求:
你的回答必须严格遵循以下格式："分期 | 理由"。
例如："T3 | 理由：报告描述肿瘤已穿透肌层，侵犯食管外膜，但未提及邻近结构受累。"
以下是需要你分析的EUS报告：........."

S1.1.2

Complete Prompt Instruction in English:

"You are a professional gastroenterologist.
Please strictly follow these criteria to determine esophageal cancer T staging, based on the 8th Edition of the UICC staging system. Carefully read and analyze the provided EUS report, then provide the most likely T stage.

T-Staging Criteria:
1. T1a:Tumor invades the lamina propria or muscularis mucosae.
2. T1b:Tumor invades the submucosa (without reaching the muscularis propria)
3. T2: Tumor invades the esophageal muscularis propria (muscle layer), but has not breached the adventitia.
4. T3: Tumor invades the adventitia.
5. T4a:Invasion of resectable adjacent structures (e.g., pleura, pericardium, diaphragm, azygous vein, peritoneum, etc.).
6.T4b:Invasion of unresectable adjacent structures (e.g., aorta, vertebrae, trachea, bronchus, lung, or other large vessels or vital organs)

Output Format Requirement:

Your response must strictly adhere to the following format: "Stage | Reason:".

For example: "T3 | Reason: The report describes the tumor penetrating the muscularis propria and invading the adventitia, with no mention of adjacent structure involvement."

Below is the EUS report for your analysis:.......”

S1.2

The text below presents the baseline instruction provided LLMs under the without-prompt experimental condition. This instruction was designed to evaluate each model’s ability to perform clinical information extraction solely from its internal knowledge, without access to detailed staging criteria or formatting guidance.

S1.2.1

Complete Instruction Without Chinese Prompts:

“你是一名专业的消化内科医生，请基于医学专业知识分析以下检查报告内容：

要求：

1. 必须明确区分T1a/T1b/T2/T3/T4a/T4b
2. 返回两列数据，用竖线分隔，示例：T1a | 肿瘤局限于黏膜固有层
3. 理由保持病理特征描述（1-2句话）

以下是需要你分析的EUS报告：.........”

S1.2.2

Complete Instruction Without English Prompts:

“You are a professional gastroenterologist. Please analyze the following medical report based on your expertise:

Requirements:

1.Clearly differentiate between T1a/T1b/T2/T3/T4a/T4b

2.Return two columns separated by vertical bars, example: T1a | Tumor confined to lamina propria

3.Justifications must describe pathological features (1-2 sentences)

Below is the EUS report for your analysis:.......”

Supplementary Section S2: Prompt Formulations for N-Staging Task

S2.1

Presented below is the full designed prompt provided to the LLMs for N-staging of esophageal cancer endoscopic ultrasound (EUS) reports in this study. This prompt was designed to ensure accurate and consistent staging performance through explicit role specification, detailed task definition, and stringent output formatting requirements.

S2.1.1

Complete Prompt Instruction in Chinese:

“你是一位专业的消化内镜医师.

请严格按照国际UICC/AJCC第8版食管癌TNM分期标准判断区域淋巴结转移（N分期）：

区域淋巴结定义：包括以下分站（需在报告中明确提及）：

1R/L（右/左下颈部/锁骨上淋巴结）

2R/L（右上/左上气管旁）

4R/L（右下/左下气管旁）
7（隆突下）
8U/M/Lo（上/中/下胸段食管旁）
9R/L（右下/左下肺韧带）
15（膈肌）
16（贲门旁）
17（胃左动脉旁）
18（肝总动脉旁）
19（脾动脉旁）
20（腹腔干旁）

N分期标准：
N0：无区域淋巴结转移
N1：1-2枚区域淋巴结转移
N2：3-6枚区域淋巴结转移
N3：≥7枚区域淋巴结转移

返回格式要求：
必须严格返回：N分期 | 理由
示例：N2 | 报告提示隆突下（7组）及胃左动脉旁（17组）共4枚淋巴结转移

以下是需要你分析的EUS报告：.........”

S2.1.2

Complete Prompt Instruction in English:

“You are a professional gastroenterologist.

"Please Strictly follow the UICC/AJCC 8th edition esophageal cancer TNM staging criteria to evaluate regional lymph node metastasis (N staging):

Definition of regional lymph nodes (explicit mention required in reports):

1. Regional Lymph Node Definition: Including the following stations (explicitly mentioned in the report):
-1R/L (Right/Left Lower Cervical/Supraclavicular）,
-2R/L (Right/Left upper paratracheal),
-4R/L (Right/Left lower paratracheal),
-7 (Subcarinal),
-8U/M/Lo (Upper/Middle/Lower paraesophageal),
-9R/L (Right/Left pulmonary ligament),
-15 (Diaphragmatic),
-16 (Pericardial),
-17 (Left gastric artery),
-18 (Common hepatic artery),
-19 (Splenic artery),
-20 (Celiac axis).

2.N Staging Criteria:
-N0: No regional lymph node metastasis.
-N1: 1–2 regional lymph node metastases.
-N2: 3–6 regional lymph node metastases.

-N3: ≥7 regional lymph node metastases.

Required Response Format:

Must strictly return: N Stage | Rationale

Example: N2 | Rationale: Report shows metastasis in 4 lymph nodes, including subcarinal (group 7) and left gastric artery (group 17).

Below is the EUS report for your analysis:.......”

S2.2

The text below presents the baseline instruction provided LLMs under the without-prompt experimental condition. This instruction was designed to evaluate each model’s ability to perform clinical information extraction solely from its internal knowledge, without access to detailed staging criteria or formatting guidance.

S2.2.1

Complete Instruction Without Chinese Prompts:

“你是一名专业的消化内科医生，请根据以下检查报告内容判断食管癌区域淋巴结转移(N分期)：

要求：

1.必须明确区分N0/N1/N2/N3

2.返回格式要求： 必须严格返回：N分期 | 理由

示例：N2 | 报告提示隆突下（7组）及胃左动脉旁（17组）共4枚淋巴结转移

以下是需要你分析的EUS报告：.........”

S2.2.2

Complete Instruction Without English Prompts:

“You are a professional gastroenterologist. Analyze the medical report content below:

Requirements:

1.Must clearly distinguish between N0/N1/N2/N3 stages.

2.Response format requirements:Must strictly return: N Stage | Rationale

Example: N2 | Report indicates 4 metastatic lymph nodes in subcarinal (Group 7) and left gastric artery (Group 17)

Below is the EUS report for your analysis:......”

Supplementary Section S3: Per-class T/N-Staging Accuracy by Model and Scenario

This section provides a detailed breakdown of the accuracy for each model on each specific stage (e.g., T1a, N1), complementing the overall accuracy metrics presented in **Tables 3 and 4** of the main manuscript. This data reveals the specific performance of each model on each specific stage under every experimental scenario, allowing for a more granular assessment. To provide a comprehensive evaluation beyond basic accuracy, we simultaneously incorporated multi-dimensional metrics: Precision, Macro-Recall, Macro-F1 score, and Quadratic Weighted Kappa (QWK). These metrics, detailed in **Supplementary Tables S3 and S4**, are critical for assessing clinical reliability, particularly in penalizing severe cross-stage misclassifications.

In the T-staging task, DeepSeek-R1 demonstrated exceptional stability across all subgroups (**Table S1**) and robust overall metrics. Specifically, under the challenging Chinese without-prompt condition, it maintained a Macro-F1 of 0.92 and a QWK of 0.97, indicating near-perfect agreement with the gold standard. In contrast, competitors such as Grok-3 exhibited a marked decline in this scenario, with Macro-F1 dropping to 0.52 and QWK to 0.66, highlighting a significant dependency on external prompt guidance.

The divergence in intrinsic reasoning capability was even more pronounced in the complex N-staging task (**Tables S2 and S4**). In the rigorous English without-prompt scenario, DeepSeek-R1 sustained a Macro-F1 of 0.80 and a QWK of 0.84. Conversely, Qwen3 experienced a catastrophic performance collapse, plummeting to a Macro-F1 of 0.19 and a QWK of 0.02, effectively resembling random guessing. The substantial disparity in QWK (0.84 vs. 0.02) confirms that DeepSeek-R1's unprompted errors are predominantly confined to clinically safer adjacent stages, whereas competitors are prone to erratic, high-risk misclassifications.

Table S1. Per-class T-staging accuracy by model across Language × Prompt scenarios

Model	T Stage	Chinese With-Prompt	Chinese Without-Prompt	English With-Prompt	English Without-Prompt
DeepSeek-R1	T1a	85.0% (68/80)	88.8% (71/80)	92.5% (74/80)	95.0% (76/80)
	T1b	98.6% (68/69)	84.1% (58/69)	76.8% (53/69)	85.5% (59/69)
	T2	94.5% (86/91)	93.4% (85/91)	94.5% (86/91)	87.9% (80/91)
	T3	98.6% (281/285)	97.5% (278/285)	94.4% (269/285)	96.5% (275/285)
	T4a	74.4% (29/39)	92.3% (36/39)	71.8% (28/39)	66.7% (26/39)
	T4b	86.9% (53/61)	93.4% (57/61)	85.2% (52/61)	59.0% (36/61)
GPT-4o	T1a	98.8% (79/80)	96.2% (77/80)	92.5% (74/80)	98.8% (79/80)
	T1b	84.1% (58/69)	72.5% (50/69)	69.6% (48/69)	58.0% (40/69)
	T2	89.0% (81/91)	50.5% (46/91)	97.8% (89/91)	28.6% (26/91)
	T3	98.6% (281/285)	97.5% (278/285)	96.8% (276/285)	93.3% (266/285)
	T4a	71.8% (28/39)	5.1% (2/39)	56.4% (22/39)	61.5% (24/39)
	T4b	93.4% (57/61)	42.6% (26/61)	83.6% (51/61)	75.4% (46/61)
Grok-3	T1a	95.0% (76/80)	100.0% (80/80)	97.5% (78/80)	97.5% (78/80)
	T1b	79.7% (55/69)	100.0% (69/69)	72.5% (50/69)	82.6% (57/69)
	T2	96.7% (88/91)	68.1% (62/91)	96.7% (88/91)	74.7% (68/91)
	T3	100.0% (285/285)	74.0% (211/285)	96.1% (274/285)	97.2% (277/285)
	T4a	74.4% (29/39)	15.4% (6/39)	66.7% (26/39)	2.6% (1/39)
	T4b	85.2% (52/61)	19.7% (12/61)	75.4% (46/61)	9.8% (6/61)
Qwen3	T1a	87.5% (70/80)	75.0% (60/80)	88.8% (71/80)	90.0% (72/80)
	T1b	94.2% (65/69)	87.0% (60/69)	87.0% (60/69)	84.1% (58/69)
	T2	95.6% (87/91)	92.3% (84/91)	96.7% (88/91)	92.3% (84/91)
	T3	93.0% (265/285)	93.0% (265/285)	96.5% (275/285)	94.7% (270/285)
	T4a	82.1% (32/39)	61.5% (24/39)	76.9% (30/39)	61.5% (24/39)
	T4b	86.9% (53/61)	55.7% (34/61)	88.5% (54/61)	57.4% (35/61)

Table S2. Per-class N-staging accuracy by model across Language × Prompt scenarios

Model	N Stage	Chinese With-Prompt	Chinese Without-Prompt	English With-Prompt	English Without-Prompt
DeepSeek-R1	N0	87.7% (107/122)	89.3% (109/122)	84.4% (103/122)	91.0% (111/122)
	N1	76.2% (112/147)	74.8% (110/147)	76.9% (113/147)	74.1% (109/147)
	N2	89.1% (179/201)	89.6% (180/201)	85.1% (171/201)	73.6% (148/201)
	N3	90.8% (99/109)	93.6% (102/109)	83.5% (91/109)	86.2% (94/109)

GPT-4o	N0	69.7% (85/122)	56.6% (69/122)	68.0% (83/122)	63.1% (77/122)
	N1	78.9% (116/147)	76.9% (113/147)	75.5% (111/147)	75.5% (111/147)
	N2	58.7% (118/201)	55.2% (111/201)	50.7% (102/201)	66.2% (133/201)
	N3	78.9% (86/109)	78.9% (86/109)	66.1% (72/109)	54.1% (59/109)
Qwen3	N0	60.7% (74/122)	71.3% (87/122)	70.5% (86/122)	5.7% (7/122)
	N1	68.7% (101/147)	72.8% (107/147)	69.4% (102/147)	5.4% (8/147)
	N2	89.1% (179/201)	85.6% (172/201)	82.6% (166/201)	97.0% (195/201)
	N3	89.0% (97/109)	91.7% (100/109)	89.0% (97/109)	0.9% (1/109)
Grok-3	N0	76.2% (93/122)	67.2% (82/122)	76.2% (93/122)	74.6% (91/122)
	N1	85.0% (125/147)	97.3% (143/147)	84.4% (124/147)	97.3% (143/147)
	N2	38.8% (78/201)	23.9% (48/201)	50.7% (102/201)	9.0% (18/201)
	N3	28.4% (31/109)	1.8% (2/109)	23.9% (26/109)	0.9% (1/109)

Table S3. Comprehensive multi-dimensional performance metrics for T-staging by model and scenario

Scenario	Model	Precision	Macro-Recall	Macro-F1	Kappa (QWK)
Chinese Without-Prompt	DeepSeek-R1	0.92	91.60%	0.92	0.97
	GPT-4o	0.92	60.70%	0.92	0.89
	Qwen3	0.81	77.40%	0.78	0.92
	Grok-3	0.63	62.90%	0.52	0.66
Chinese With-Prompt	DeepSeek-R1	0.91	89.70%	0.9	0.98
	GPT-4o	0.91	89.30%	0.9	0.98
	Qwen3	0.88	89.90%	0.88	0.97
	Grok-3	0.89	88.50%	0.89	0.97
English Without-Prompt	DeepSeek-R1	0.87	81.80%	0.83	0.94
	GPT-4o	0.87	69.30%	0.83	0.92
	Qwen3	0.85	80.00%	0.81	0.92
	Grok-3	0.75	60.70%	0.61	0.87
English With-Prompt	DeepSeek-R1	0.87	85.90%	0.86	0.96
	GPT-4o	0.87	82.80%	0.86	0.95
	Qwen3	0.9	89.10%	0.9	0.97
	Grok-3	0.87	84.20%	0.86	0.94

*Macro-metrics represent the unweighted mean of per-class scores. QWK quantifies agreement by heavily penalizing severe cross-stage misclassifications.

Table S4. Comprehensive multi-dimensional performance metrics for N-staging by model and scenario

Scenario	Model	Precision	Macro-Recall	Macro-F1	Kappa (QWK)
Chinese Without-Prompt	DeepSeek-R1	0.88	86.80%	0.87	0.9
	GPT-4o	0.88	66.90%	0.87	0.77
	Qwen3	0.82	80.30%	0.81	0.83
	Grok-3	0.68	47.60%	0.42	0.56
Chinese With-Prompt	DeepSeek-R1	0.87	86.00%	0.86	0.9
	GPT-4o	0.87	71.60%	0.86	0.81
	Qwen3	0.82	76.90%	0.78	0.79
	Grok-3	0.7	57.10%	0.57	0.72
English Without-Prompt	DeepSeek-R1	0.8	81.20%	0.8	0.84
	GPT-4o	0.8	64.70%	0.8	0.73
	Qwen3	0.42	27.20%	0.19	0.02
	Grok-3	0.63	45.40%	0.38	0.52
English With-Prompt	DeepSeek-R1	0.85	82.50%	0.84	0.87
	GPT-4o	0.85	65.10%	0.84	0.78
	Qwen3	0.8	77.90%	0.79	0.81
	Grok-3	0.69	58.80%	0.58	0.73

*Macro-metrics represent the unweighted mean of per-class scores. QWK quantifies agreement by heavily penalizing severe cross-stage misclassifications.

Supplementary Section S4: Confusion Matrices

This appendix provides supplementary data in the form of confusion matrices to detail each model's performance across experimental conditions. A confusion matrix serves as a visualization tool for evaluating classification models by contrasting the gold standard ("Reference") against model predictions, thereby illustrating both overall accuracy and specific error patterns.

In each matrix, main-diagonal cells represent correctly classified cases, where darker shading and higher values indicate greater predictive accuracy for that class. Off-diagonal cells represent misclassifications. Analyzing their distribution not only helps assess overall accuracy but also reveals error tendencies—particularly important for ordinal classifications like T/N staging, where error severity correlates with "misclassification distance": the farther from the main diagonal, the more clinically severe the cross-stage error typically is.

For cross-model comparison, each model is presented under four conditions: Chinese/English × with designed prompt/without designed prompt. This study reveals that unprompted and cross-lingual scenarios posed greater challenges; DeepSeek-R1 maintained relative stability under these conditions, while other models exhibited more pronounced off-diagonal distributions.

S3.1

The figures below show the confusion matrices for the DeepSeek-R1 model under different conditions. The model's predictions are highly concentrated along the main diagonal, indicating very high accuracy. Most residual errors occur between adjacent stages (e.g., N1 vs N2), a clinically more acceptable pattern that further demonstrates the model's stability and reliability.

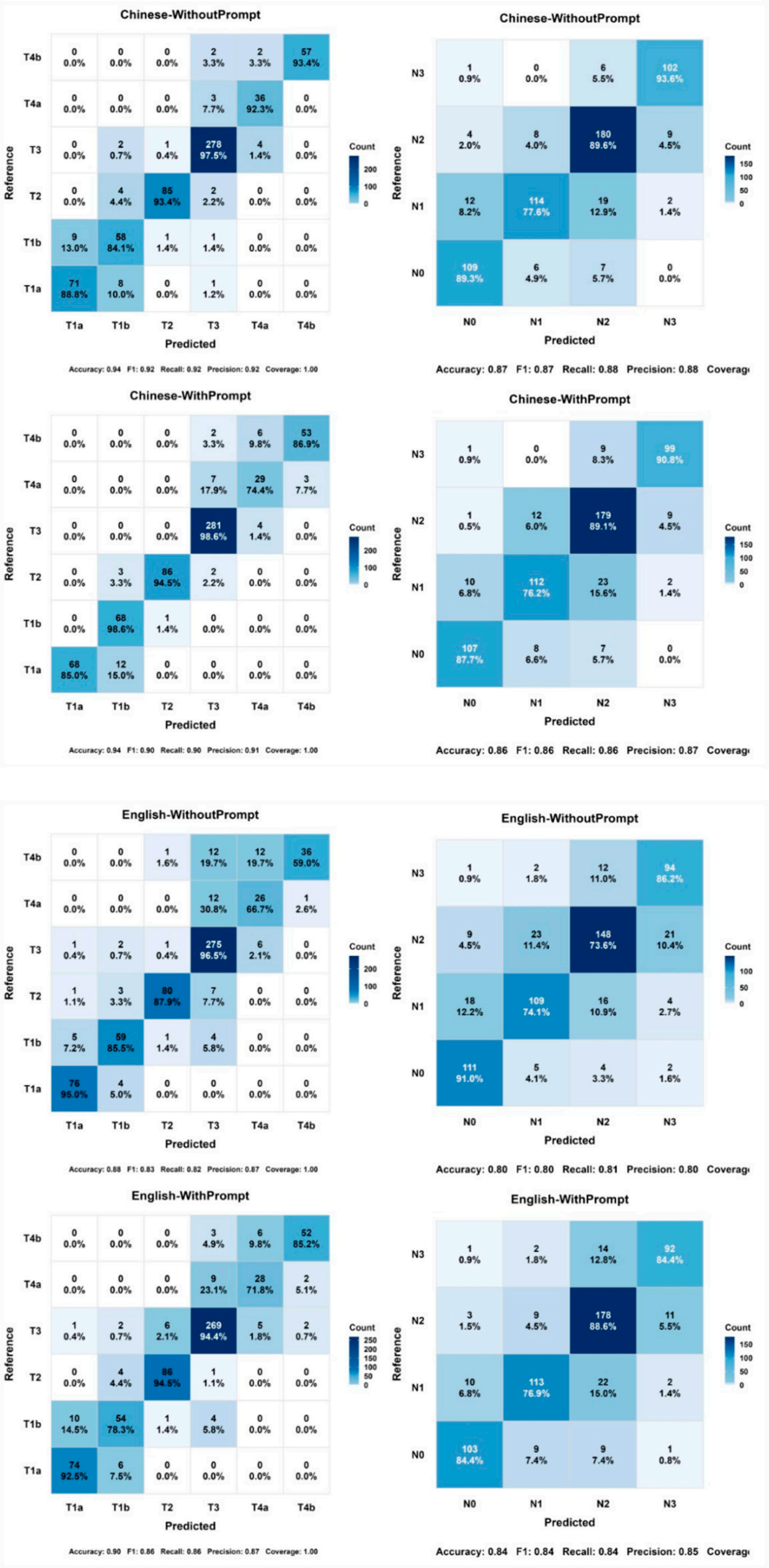

Figure S1. DeepSeek-R1 Confusion Matrices

S3.2

The figures below show the confusion matrix performance of the GPT-4o model in T and N-staging tasks. While its performance was close to DeepSeek-R1 when prompts were provided, off-diagonal cells increased substantially without prompt—particularly in Chinese tasks. For instance, under the "Chinesewithout-prompt" N-staging condition, the model showed frequent misclassifications between N0 and N1 categories, indicating a strong dependency on prompt guidance.

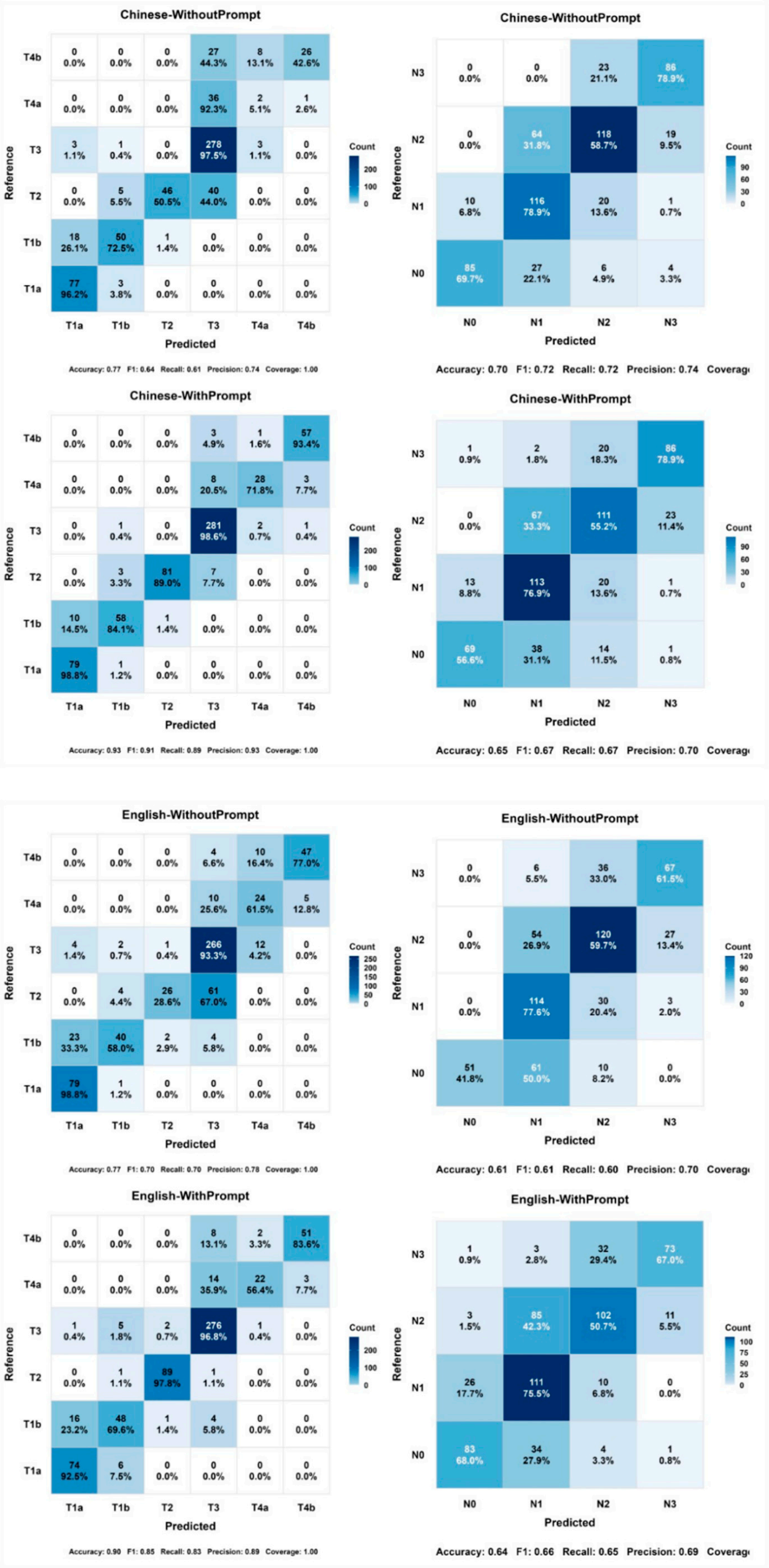

Figure S2. GPT-4o Confusion Matrices

S3.3

The figure below presents the confusion matrix performance of the Grok-3 model. Under prompted conditions, predictions demonstrate tight concentration along the main diagonal; however, prompt removal leads to pronounced off-diagonal dispersion accompanied by marked performance degradation—particularly evident in Chinese-without-prompt T-staging. Across multiple N-staging tasks, the model exhibits more frequent severe cross-stage misclassifications. Collectively, these results indicate Grok-3's high dependency on external prompts and substantially diminished predictive reliability in the absence of explicit instructions.

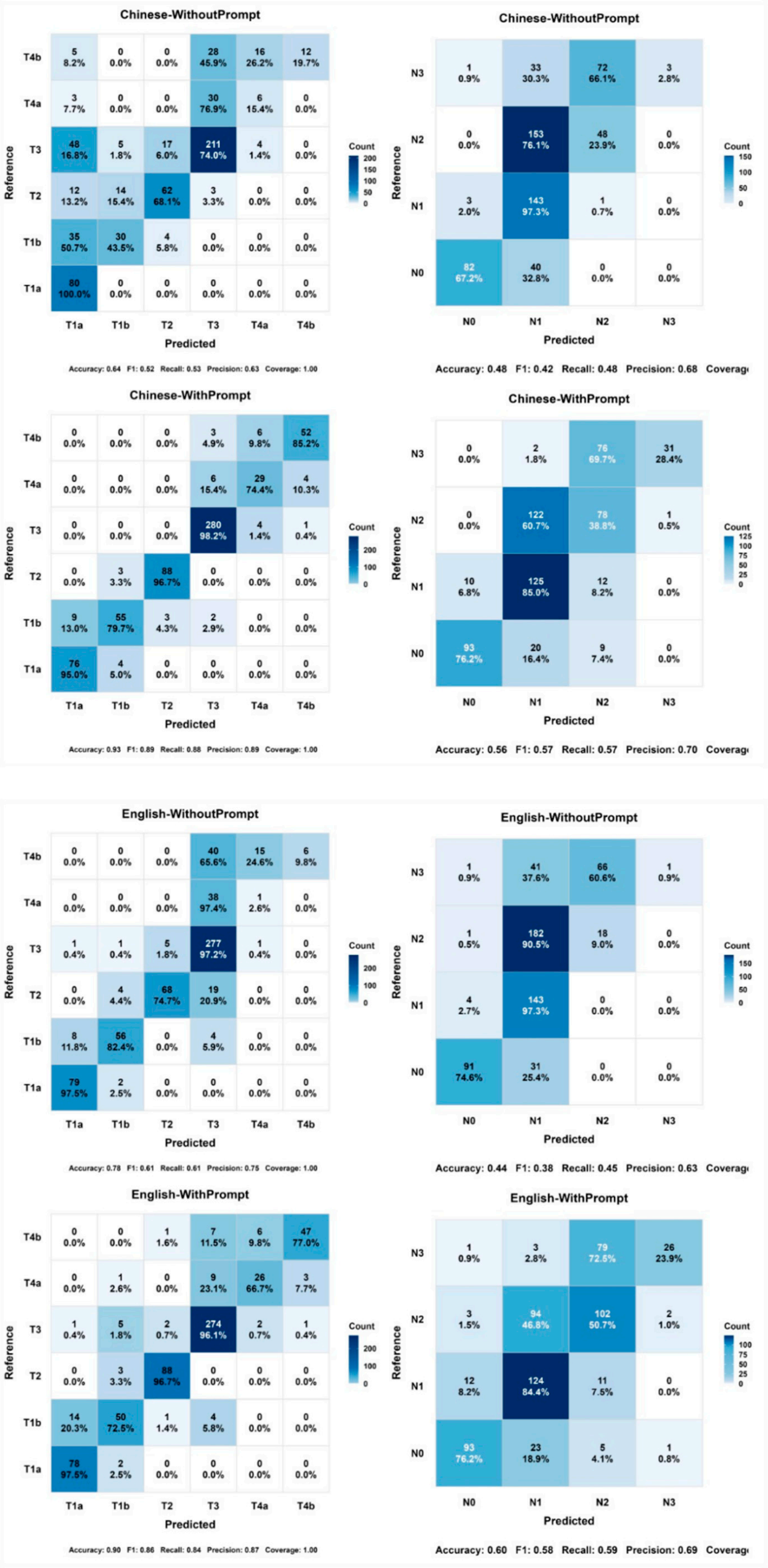

FigureS3. Grok-3 Confusion Matrices

S3.4

The figures below show the confusion matrix performance of the Qwen-3 model. It demonstrates strong performance in most T-staging tasks with predictions concentrated along the main diagonal; however, N-staging proves more challenging. Notable confusion appears among N1, N2, and N3 categories in the Chinese with-prompt condition, while the most pronounced off-diagonal spread and performance decline occur in Englishwithout-prompt N-staging. These patterns highlight cross-lingual unprompted scenarios as a key weakness for this model.

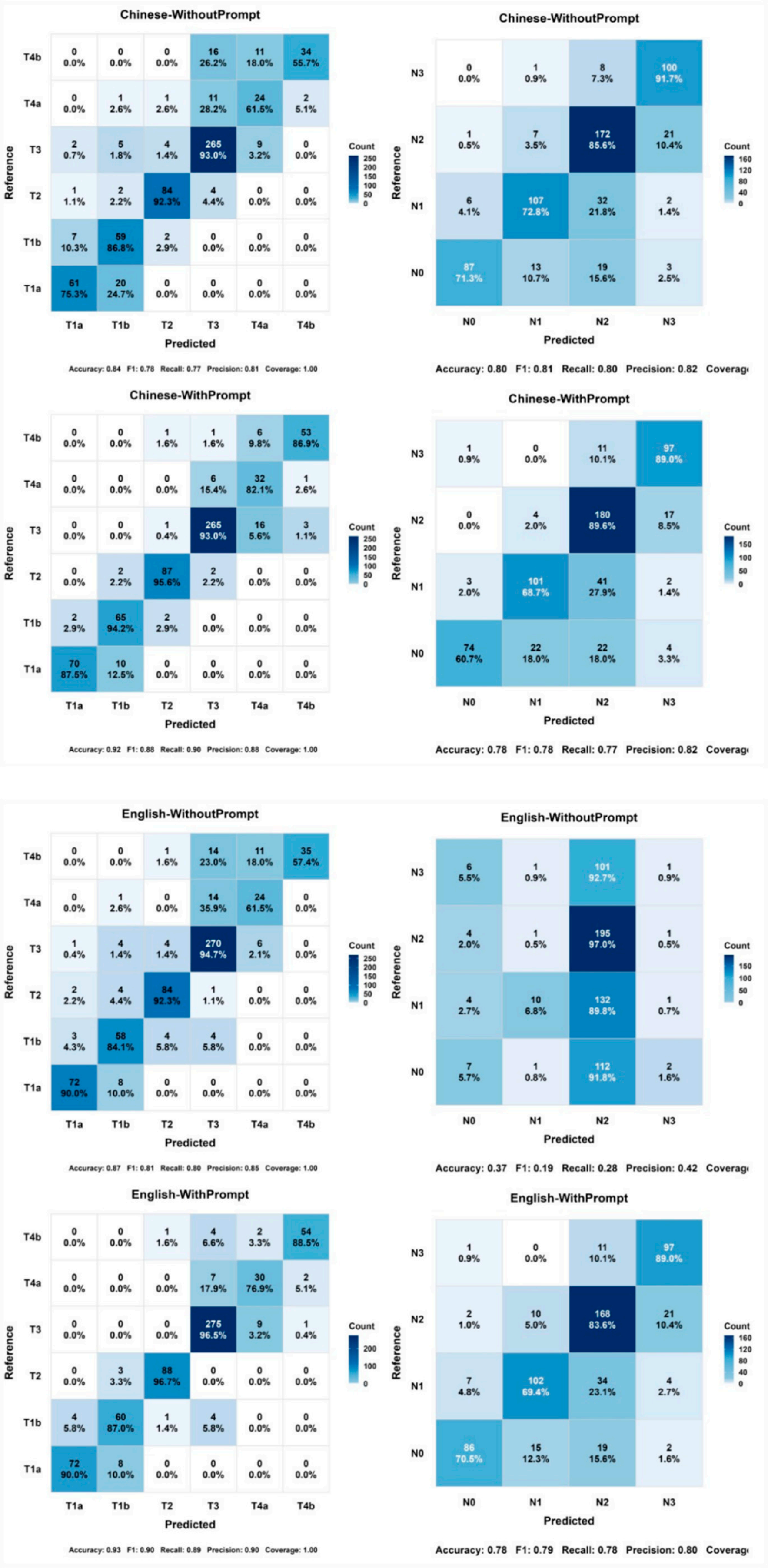

Figure S4. Qwen3 Confusion Matrices
